# Current genetic strategies to investigate gene functions in *Trichoderma reesei*

**DOI:** 10.1186/s12934-023-02104-3

**Published:** 2023-05-10

**Authors:** Chixiang Ma, Jialong Liu, Jiaxin Tang, Yuanlu Sun, Xiaojie Jiang, Tongtong Zhang, Yan Feng, Qinghua Liu, Lei Wang

**Affiliations:** 1 China Medical University-The Queen’s University of Belfast Joint College, Shenyang, Liaoning, 110122 China; 2 College of Life Sciences, Shanxi Agricultural University, Jinzhong, 030801 Shanxi China; 3 College of Basic Medical Sciences, Shanxi Medical University, Taiyuan, 030001 Shanxi China

**Keywords:** *Trichoderma reesei*, Non-homologous end joining, Homologous recombination, RNA interference, Promoter replacement, Repeat-induced point mutation, CRISPR/Cas9, Gene complementation and overexpression

## Abstract

The filamentous fungus *Trichoderma reesei* (teleomorph *Hypocrea jecorina, Ascomycota*) is a well-known lignocellulolytic enzymes-producing strain in industry. To increase the fermentation titer of lignocellulolytic enzymes, random mutagenesis and rational genetic engineering in *T. reesei* were carried out since it was initially found in the Solomon Islands during the Second World War. Especially the continuous exploration of the underlying regulatory network during (hemi)cellulase gene expression in the post-genome era provided various strategies to develop an efficient fungal cell factory for these enzymes’ production. Meanwhile, *T. reesei* emerges competitiveness potential as a filamentous fungal chassis to produce proteins from other species (e.g., human albumin and interferon α-2b, SARS-CoV-2 N antigen) in virtue of the excellent expression and secretion system acquired during the studies about (hemi)cellulase production. However, all the achievements in high yield of (hemi)cellulases are impossible to finish without high-efficiency genetic strategies to analyze the proper functions of those genes involved in (hemi)cellulase gene expression or secretion. Here, we in detail summarize the current strategies employed to investigate gene functions in *T. reesei.* These strategies are supposed to be beneficial for extending the potential of *T. reesei* in prospective strain engineering.

## Background

Plant biomass which mainly comprises cellulose, hemicellulose, and lignin, is one of the most abundant renewable energy sources on the earth [1]. The biodegradation of plant biomass represents a crucial step in the biorefinery industry and this process relies on the hydrolysis ability of lignocellulolytic enzymes to plant biomass substrates [2–4]. The filamentous fungus *T. reesei* strain QM6a was isolated from the Solomon Islands during the Second World War because of its destruction to army textiles, such as cotton-based tents and clothing [5]. As it turns out that *T. reesei* can secret various saccharification enzymes required for biomass degradation. Hence, engineering of *T. reesei* QM6a has been proceeding for high yield of lignocellulolytic enzymes to reduce industrial costs by physical (ultraviolet) or chemical (nitrosoguanidine, 2-deoxyglucose) mutagenesis and rational genetic modification [6–10]. In the 1970s, random mutagenesis was the primary method to obtain high-yield strain of lignocellulolytic enzymes, and some famous strains, such as RUT-C30 and QM9414, have been isolated and used till now [6, 11]. Penttilä et al. developed a transformation system in 1987 that initiated the era of genetic engineering in *T. reesei* [12]. The whole genome sequencing project in 2008 accelerated the development of novel genetic manipulation strategies for investigating gene functions in *T. reesei* QM6a and its derived strains. Based on these strategies and identified regulators of (hemi)cellulase gene expression, further strain engineering was carried out for high-yield (hemi)cellulases [13–17]. It was documented that *T. reesei* cellulase secretes up to 100 g/L in the extracellular space [18, 19]. Nevertheless, the underlying mechanism for regulating the (hemi)cellulase gene expression is not completely understood yet. Therefore, developing novel and efficient genetic strategies to investigate the gene functions will contribute to future strain engineering in *T. reesei.*

Among these genetic strategies for investigating gene functions, the most common one is to weaken or strengthen gene expression of a specific gene. However, gene knockout or knockdown strategy seems to be more accurate than gene overexpression when investigating a function of a specific gene. The reason is that too high expression of functional proteins in host cells could not lead to a distinct phenotype with parent strain due to already oversaturated protein level [20]. Sometimes, gene overexpression will lead to a similar phenotype as the knockout of the same gene, disturbing the correct prediction of gene functions [21]. Moreover, an expression cassette containing the promoter, open reading frame (ORF), terminator, and selection marker is usually required and is introduced into the genome during a gene overexpression, which may result in unwanted phenotype in case of random integration event [22]. In this review, we mainly summarize the current aspects of genetic strategies applied to investigate gene functions in *T. reesei*, including selection markers, gene knockout, gene knockdown, repeat-induced point mutation (RIP), Cas9-mediated gene editing, gene complementation, and overexpression. Our outlined strategies are also a valuable reference for functional gene studies in other fungal species, especially in *Trichoderma* spp. Of course, the development of transformation methods is another critical aspect of the reaserach of gene functions in *T. reesei*. For the extensive review on transformation methods, including PEG-mediated transformation of protoplasts, agrobacterium tumefaciens-mediated transformation, biolistic transformation, and electroporation, we direct the readers to the excellent review summarized by Tomico-Cuenca et al. [23].

## Selection markers

To avoid false positive events during transformant screening, a faithful selection marker should be selected and used when the genetic strategies are applied to investigate gene function in *T. reesei*. All the selection markers functioned in *T. reesei* are listed in Table 1. Auxotrophic markers usually derive from *T. reesei* itself and encode an enzyme that is indispensable for survival of strains at specified culture conditions. Of note is a *suc1* gene originating from *Aspergillus niger*, which also has been successfully applied in *T. reesei* [24]. The *suc1* encodes an invertase that is lacking in *T. reesei* and it can confer the *T. reesei* to utilize sucrose as a sole carbon source [24]. Most antibiotic markers originate from other species, such as *Escherichia coli*, *Streptomyces hygroscopieus*, *Streptoalloteichus hindustanus*, *Aspergillus nidulans*, *Aspergillus oryzae*, etc. [12, 25–31]. They can either confer corresponding resistance against an appointed chemical reagent that suppresses the growth of parental strains at a suitable concentration or enable the effective utilization of a non-metabolizable nutrient substance.

Sometimes, to clarify the gene functions in detail, two or more genes should be operated orderly in *T. reesei*. One way to do that is selecting and using different selection markers when the target genes are modified in turn. Another way to achieve that is marker recycling, which allows reuse of selection markers in next sequential gene modification. Several different approaches have been developed to achieve marker recycling in *T. reesei* and also summarized by Tomico-Cuenca et al.[23].

**Table 1:** Selection markers used in *T. reesei*

Gene	Encoded protein	Source	Refs.
**Auxotrophic markers**			
*pyr4*	orotidine-5-monophosphate decarboxylase	*Trichoderma ressei*	[32]
*pyr2*	orotate phosphoribosyl transferase	*Trichoderma ressei*	[33]
*asl1*	argininosuccinate lyase	*Trichoderma ressei*	[34]
*ade2*	phosphoribosylaminoimidazole carboxylase	*Trichoderma ressei*	[33]
*suc1*	invertase	*Aspergillus niger*	[24]
*hxk1*	hexokinase	*Trichoderma ressei*	[25]
*hem8*	ferrochelatase	*Trichoderma ressei*	[26]
**Antibiotic markers**			
*hph*	hygromycin B phosphotransferase	*Escherichia coli*	[27]
*amds*	acetamidase	*Aspergillus nidulans*	[12]
*argB*	ornithine carbamoyltransferase	*Aspergillus nidulans*	[12]
*nptII*	neomycin phosphotransferase II	*Escherichia coli*	[28]
*bar*	phosphinothricin acetyltransferase	*Streptomyces hygroscopieus*	[29]
*ptrA*	thiamine thiazole synthase	*Aspergillus oryzae*	[30]
*ble*	bleomycin binding protein	*Streptoalloteichus hindustanus*	[31]

## Homologous recombination-mediated gene knockout

Genomic integrity and faithful replication are crucial for avoiding mutations that may lead to cells out-of-control or death [35, 36]. Several mechanisms have evolved to protect the genome against DNA damage resulting from ultraviolet (UV) rays, reactive oxygen species (ROS), virulent chemicals, etc. [37, 38]. Homologous recombination (HR) and non-homologous end joining (NHEJ) are the two main pathways to repair DNA damage in case of double-strand breaks (DSBs) [39–41]. In eukaryotic organisms, in addition to functions in DSB repair, the NHEJ and HR pathways also determine how cells integrate foreign DNA [42]. Random integration of foreign DNA is dominant in host cells and resultantly suppresses homologous genomic integration, leading to very low efficiencies of gene targeting [43, 44]. In *T. reesei*, some strategies have been adopted to facilitate HR during gene targeting: (i) extending homologous arm; (ii) suppressing NHEJ pathway; and (iii) introducing I-*Sce*I nuclease to produce artificial DSBs.

### Extending homologous arm

For efficient gene knockout events in *T. reesei*, approximately 500 ~ 1500 bp homologous sequences flanking the selection marker at 5’ and 3’ regions are indispensable to construct DNA cassettes [45]. However, this strategy usually exhibits a low recombination rate (5%~10%) at the desired position [45]. Considering the long homologous arm is beneficial to facilitate the HR probability, more than 2000 bp homologous sequence was employed in gene knockout events in some tests [46–48]. Even so, the increment in HR efficiency does not seem to be remarkable. Moreover, the long homologous arms have increased the difficulty in synthesization, as well as lower transmembrane transport efficiency during fungal transformation. It even hinders searching appropriate restriction sites at the DNA cassette for its linearization before the transformation.

### Suppressing NHEJ pathway

**Fig. 1:**
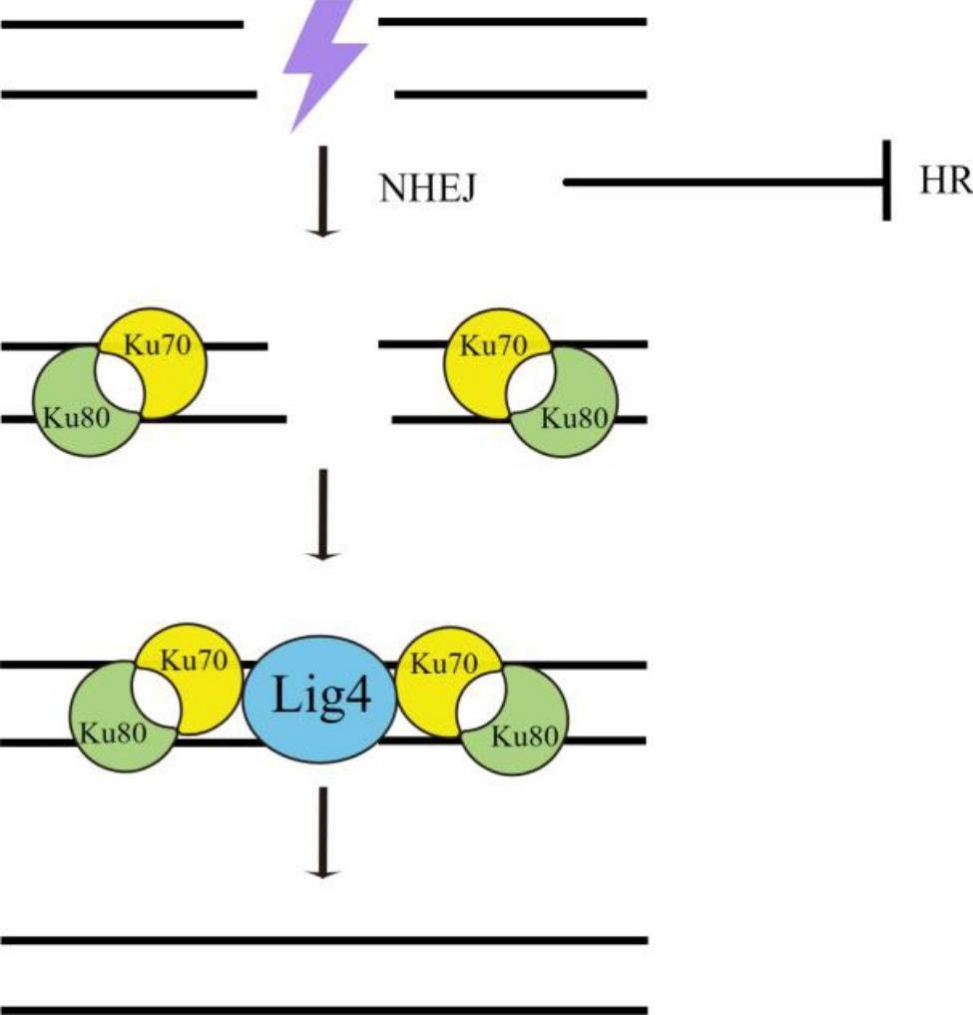
**NHEJ pathway suppresses HR pathway in*T. reesei.*** NHEJ starts with recognizing and binding the DSBs by the Ku70/80 complex, which serves as a scaffold to recruit repair enzymes Mus53(Lig4) to finish the DNA ends ligation. Because NHEJ pathway is dominant during DSB repair in eukaryotic organisms including *T. reesei*, the NHEJ defective strains, such as Δ*ku70* and Δ*mus53*, have been developed to increase the rate of HR in *T. reesei*

Given the NHEJ pathway is dominant during DSB repair in eukaryotic organisms, its suppression will improve the HR efficiency [49]. NHEJ starts with recognizing and binding the DSBs by the Ku70/80 complex, which serves as a scaffold to recruit repair enzymes such as Mus53 (orthologs of human Lig4) to finish the DNA ends ligation (Fig. 1). To increase the rate of HR events during transformation in *T. reesei*, the NHEJ defective strains, such as Δ*ku70* and Δ*mus53*, were developed to avoid ectopic DNA integration [26, 50]. In these NHEJ defective strains, the HR-mediated gene knockout not only needs a shorter homologous arm but also shows a significant increase in gene targeting rate (> 95%) [45]. The latest findings showed that light can upregulate the *ku70* expression, implying a dark environment may be favorable for higher HR efficiency [26].

Even though the HR efficiency is tremendously increased in NHEJ defective strain, it remains low transformation frequency in many fungi, making it arduous to obtain enough transformants for further screening [51]. Worse, loss of NHEJ affects genomic stability and leads to the accumulation of spontaneous mutations [52–54]. Given this, multiple actions were taken to overcome deficiency. For example, a transient silencing of *mus53* or *ku80* using small interfering RNA (siRNA) was performed to improve targeted gene integration (*mus53*, 59%; *ku80*, 37%) in *T. reesei* M44 strain that derived from the original QM6a isolate [55]. In other cases, the NHEJ defect was subsequently removed following the gene targeting to avoid the above mentioned negative effect by crossing of mutants with a sexually competent strain [52, 56].

### Expression of I-*Sce*1 endonuclease

To develop alternative methods for efficient gene targeting, the I-*Sce*I endonuclease encoded by the *Saccharomyces cerevisiae* mitochondrial genome was introduced to artificially produce DSB in *T. reesei* JP7.7 reportor strain [51, 57]. JP7.7 was developed from the mutant strain, RL-P37, via several gene modification steps, and two 18 bp I-*Sce*I restriction sites (5′-TAGGGATAACAGGGTAAT-3′) were designed to incorporate its genome [51, 57]. When a donor DNA flanked by regions homologous to the surrounding locus of DSB was present, the DSB can be rapidly and efficiently repaired. This method not only increases the number of transformants during transformation but also promotes the efficiency of targeted deletion of the interested gene [51, 57]. Meanwhile, combining the I*-Sce*I enzyme-mediated integration with the *ku70* mutant resulted in up to 100% HR efficiency at the appointed integration site [58].

## Gene knockdown

Gene knockout strategy involves the complete erasing of target genes or inactivating them through nonsense mutations whereas gene knockdown leads to mRNA degradation or declining RNA synthesis. In the genome, there are numerous genes crucial for cell survival or fundamental growth, of which knockout is very difficult or impossible, hampering functional gene analyses [20]. To solve this problem, there are two strategies developed in *T. reesei*, (i) RNA interference (RNAi) and (ii) local promoter replacement, to control gene expression abundance for further functional studies.

### RNA interference

**Fig. 2:**
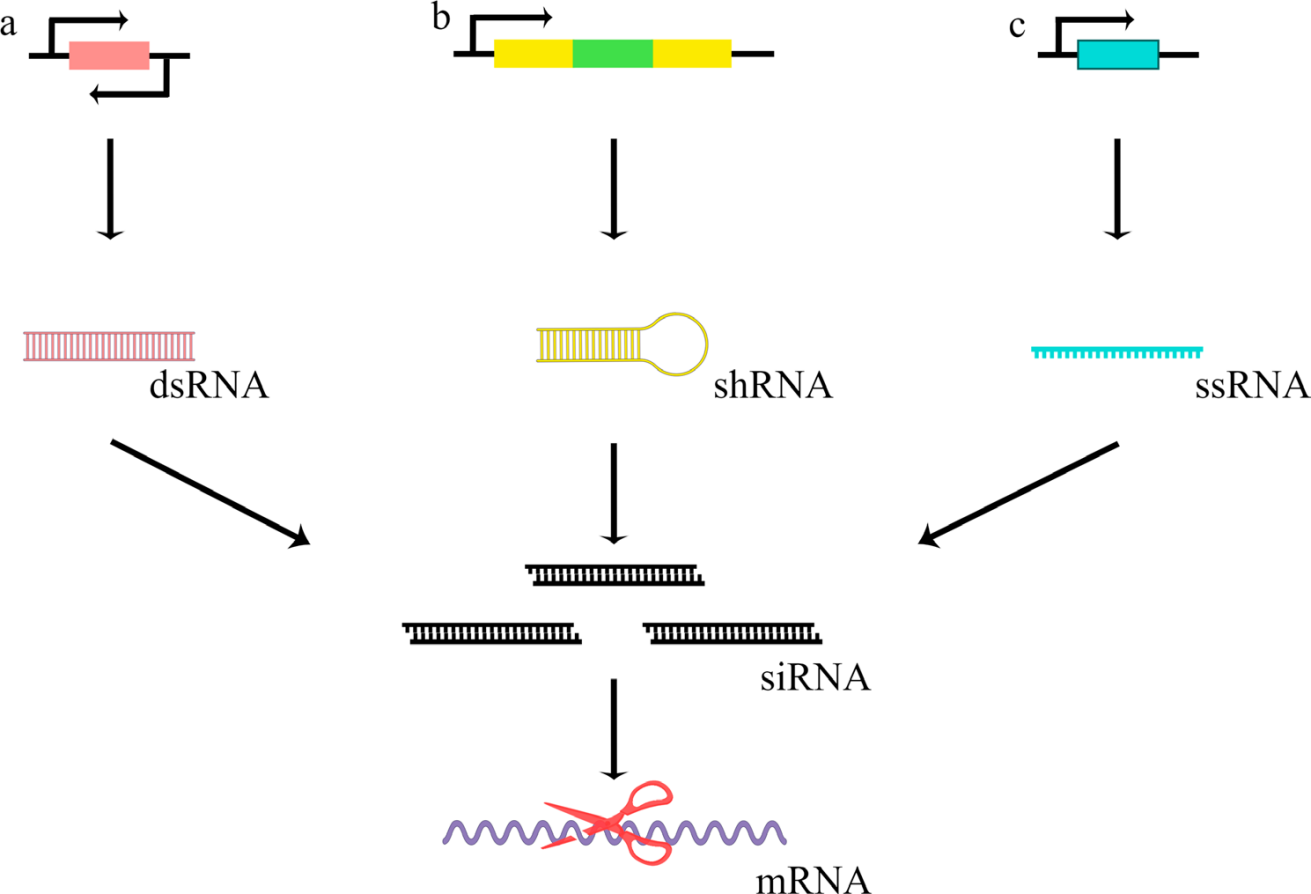
**Strategies for synthesis of siRNA in*T. reesei***. (a) Identical sequences with target genes are transcribed in both directions under the control of a dual promoter to generate dsRNA. (b) The shRNA is generated by constructing a cassette in which the promoter drives the transcription of two inverted repeat sequences identical to the target gene with a spacer. (c) A complementary sequence to targeting mRNA is transcribed as antisense ssRNA by constructing a cassette in which the promoter drives a transcription of sequence from the antisense strand. The antisense ssRNA will pair with target mRNA to generate the dsRNA. The dsRNA as well as shRNA will be processed by ribonuclease Dicer to generate siRNA for RNAi induction

RNA interference refers to a conserved process induced by double-stranded RNA (dsRNA) to specifically degrade the homologous mRNA in most eukaryotic cells [59–61]. The RNAi system has been successfully applied in dozens of filamentous fungi including *T. reesei* (Fig. 2) to investigate the functions of the interested genes by generating specific siRNA, which triggers the degradation of target mRNA [62, 63]. In *T. reesei*, several methods are employed to produce the dsRNA or the short hairpin-RNA (shRNA) in vivo [64, 65]. Whereafter, the dsRNA or shRNA is processed by ribonuclease Dicer to generate siRNA. The dsRNA usually is generated by a plasmid in which a dual promoter is used to transcribe the identical DNA sequences with target genes in both directions [65]. However, only a promoter is needed to generate the shRNA because it drives the transcription of two inverted repeat sequences identical to the target gene with a spacer [64]. Besides, in some cases, only an antisense single-stranded RNA (ssRNA) is transcribed using an antisense strand as a template to form the dsRNA with mature mRNA for further induction of RNAi [66].

In 2009, Brody et al. first achieved the cellobiohydrolase II gene (*cbh2*) knockdown in *T. reesei* Rut-C30 by expressing a *cbh2*-specific shRNA in virtue of the endogenous cellobiohydrolase I gene (*cbh1*) promoter [64]. Northern blot analysis of small siRNAs, quantitative RT-PCR (RT-qPCR) of *cbh2* mRNA, and sodium dodecyl sulfate-polyacrylamide gel electrophoresis (SDS-PAGE) analysis of Cbh2 proteins adequately proved the feasibility of the RNAi system in *T. reesei* [64]. A similar strategy was also applied in *T. reesei* metabolic engineering to increase xylitol production by silencing the D-xylulokinase (*xyiH*) gene [31]. Schmoll et al. achieved gene knockdown of *gna3* encoding a G-alpha subunit of the G-protein/cyclic AMP signaling pathway in *T. reesei*. In this case, an antisense ssRNA of *gna3* mRNA was expressed using the constitutive *gpd1* promoter [66]. To utilize the secretion capacity of the *T. reesei* host to express a recombinant lipase, the endogenous *cbh1* was knockdown by expressing a hairpin dsRNA using its own promoter [67]. These dsRNAs can specifically trigger *cbh1* mRNA degradation to provide more secretion space for recombinant protein, resulting in a 1.8- to 3.2-fold increase in heterologous lipase production [67]. He et al. constructed an RNAi system in which two target genes were simultaneously knocked down using dual promoters derived from *T. reesei* (*rp2* promoter) and *Aspergillus nidulans* (*trpC* promoter) [68]. Gao et al. achieved the high-efficiency silencing of *Trcot1* involved in hyperbranched phenotype by expressing a dsRNA with two promoters (*pdc1* and *eno1*) in a head-to-head manner [65]. However, due to promoter features, these RNAi systems were either uncontrolled or only applicable under cellulose-induced conditions.

In 2015, a copper-responsive promoter (P_*tcu1*_) was identified in *T. reesei* [69]. The expression activities of P_*tcu1*_ are unlimited without copper ions in the media and are effectively inhibited by copper ions in a concentration of more than 500 nM [69, 70]. Based on this promoter, we developed a copper-controlled RNAi system in *T. reesei* QM9414 and TU-6 mutants. In this system, the shRNA was expressed in the condition without copper while repressed when the copper was present [71]. This RNAi system allows investigating gene functions independent of nutritional states and mimicking the gene complementation easily by including copper in the media to exclude negative effects that may result from the random insertion of the shRNA expression cassette into the genome [72]. Using the copper-responsive RNAi system, the functions of several genes that could not be easily deleted by HR strategy were clarified, such as *rxe1*, *cyc8*, and *acf1* [21, 73, 74], indicating RNAi is a promising tool for characterizing functions of target genes in *T. reese*i.

### Local promoter replacement

For those genes that are crucial for survival or fundamental growth, promoter replacement engineering based on tunable promoters is alternative method for conditional control of gene expression levels [75]. It ensures normal gene expression during genetic transformation and screening for obtaining the transformants, whereas gene expression is closed by manipulating the transcriptional activity of promoters for further functional analysis.

**Fig. 3:**
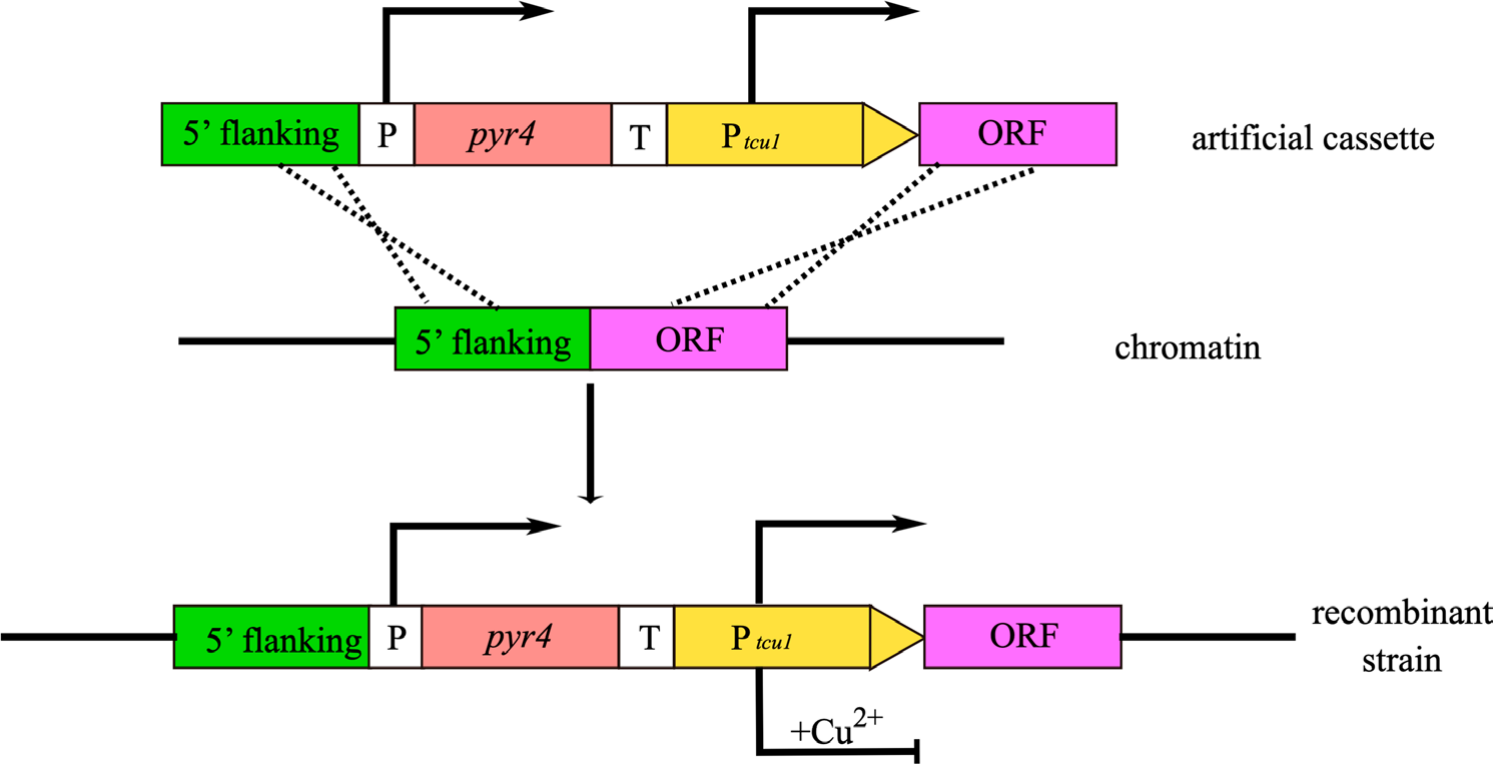
**Strategies for local promoter replacement.** An artificial cassette was orderly assembled in vitro by four fragments containing 5’ flanking region, the *pyr4* expression cassette (P and T represent promoter and terminator, respectively), P_*tcu1*_ promoter, and ORF of the local gene. When an artificial cassette integrates into the correct loci of the genome, the local gene expression will be controlled by copper from the environment

In *T. reesei*, Zheng et al. established a P_*tcu1*_-based promoter replacement system in which the intact *pyr4* expression cassette was followed by a tunable P_*tcu1*_ promoter [20]. Meanwhile, the gene body from the initiation codon ATG and its upstream sequence served as homologous arms, respectively, achieving effective integration at the target position (Fig. 3) [20]. As a proof-of-concept, the function of two putative Spt-Ada-Gcn5 acetyltransferase (SAGA) complex subunits (Gcn5 and Ada2)-encoding genes were investigated by reducing their expression during cultivation with copper [20, 76]. On the contrary, this system could analyze the phenotype of gene complementation by removing the copper. In recent studies, the more mysterious functions of novel genes, especially those essential genes that affected growth or spore formation, were revealed by this strategy [21, 73].

It is well known that *T. reesei* (hemi)cellulase gene expression is strictly induced by cellulose, sophorose, lactose, etc., and repressed by glucose [77]. Therefore, these carbon source-dependent promoters from (hemi)cellulase genes seem to be adaptive for functional investigation of target genes via local promoter replacement when the *T. reesei* strain is cultivated with glucose as a carbon source [78]. In this case, the expression of target genes will be repressed due to extremely low transcriptional activity of (hemi)cellulase promoters in glucose-containing media. However, these carbon source-dependent promoters are not applied to knock down the target genes via promoter replacement strategy in the inducible carbon source because of their inherent high transcriptional capacity at this condition [79]. In addition, some constitutive promoters with weak transcriptional activity are also applicable to replace native promoters of interested genes for further functional studies, especially during the studies of those biological functions extremely sensitive to protein abundance.

## Repeat-induced point mutation

**Fig. 4:**
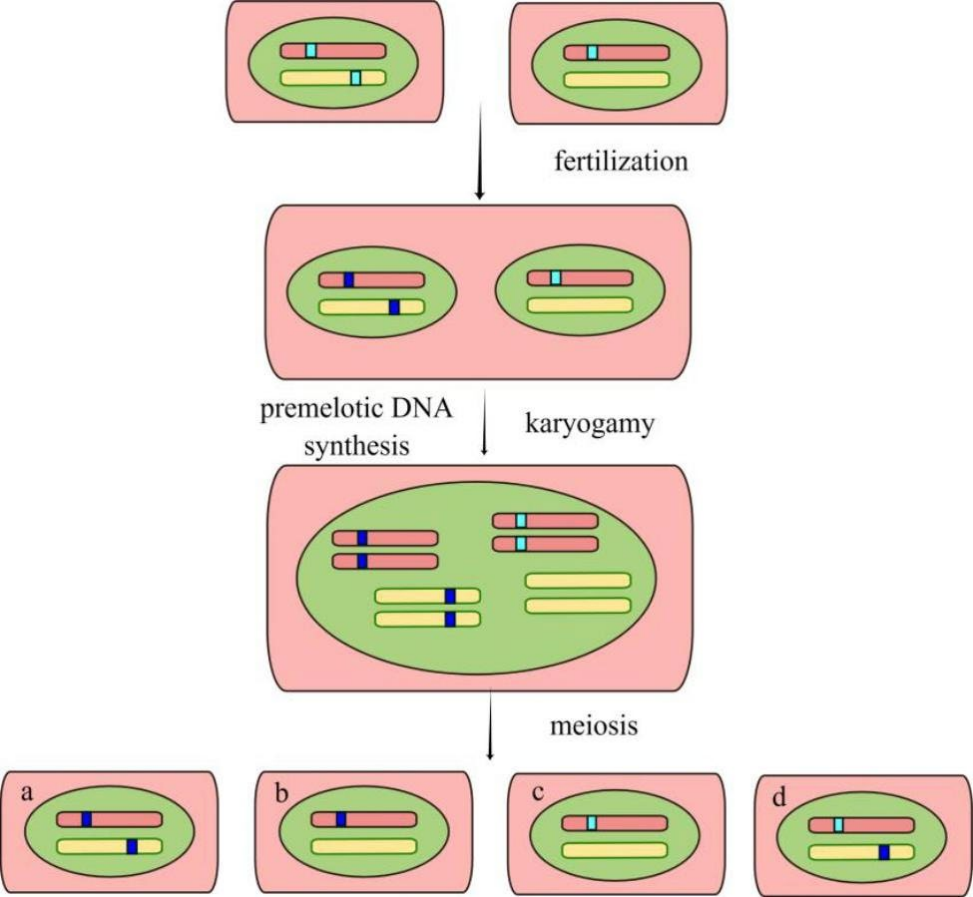
**Diagram of RIP process.** Two haploid strains with different mating types are crossed. Between them, a strain (left) harboring an unlinked duplication (box) of the chromosomal fragment. For clarity, only two chromosomes are indicated. After fertilization, both copies of the strain harboring duplicated fragments are mutant by RIP during the dikaryon stage. Karyogamy and meiosis immediately follow and the four possible combinations (a-d) of chromosomes in progeny are shown. If the duplicated fragment derives from the target gene coding region, the target gene of two progenies (a-b) is inactivated

RIP as a genome defense system against transposable elements or repetitive sequences was originally discovered in the *Neurospora crassa* [80–82]. RIP is a premeiotic procedure that ascertains the repetitive sequence and then produces the G/C to A/T base pair mutations either within or adjacent to the regions of repetitive DNA (Fig. 4) [83]. Genome-wide scan and pairing between repetitive sequences were two crucial factors in determining the RIP frequency which may be affected by accessibility between repetitive sequences [84]. RIP occurrence was effortless on tandem duplication compared with unlinked duplication [85].

Up to now, RIP has been confirmed by experiments or in silico analyses in many Pezizomycotina fungi including *T. reesei* [86, 87]. In some fungi, RIP was thought to be dependent on C5-cytosine methylation despite the detailed mechanism of how C5-cytosine methylation leads to C-to-T transform is still unclear [88, 89]. In the *N. crassa* genome, two putative DNA C5 cytosine methyltransferases, Rid, and Dim2 trigger RIP and show evident dinucleotide preference (CpA > CpT > CpG > CpC) [90, 91]. These two methyltransferase orthologs also exist in *T. reesei* and exhibit a different dinucleotide preference (CpG ≥ CpA≫CpT > CpC) [83].

On account of the C-to-T mutation, both repetitive sequences will generate AT-rich blocks in which the stop codons are frequent [92]. RIP has been developed as a molecular tool to study gene functions by early terminating translation or converting the amino acid sequence of the encoded protein. Numerous evidences, especially in *N. crassa*, confirmed this possibility [92, 93].

In 2017, Li et al. verified that the RIP occurred in *T. reesei* by sexual crossing between strains containing wild-type CBS999.97 and its derived strains (Δ*blr1*, Δ*env1*, Δ*ku70*, and Δ*env1&*Δ*ku70*) [83, 86]. In these strains, the hygromycin-resistant (*hph*) gene served as a repetitive sequence to induce the RIP before the premeiotic stage. RIP was proved by facts that numerous mutations occur in repetitive *hph* genes regardless of being physically adjacent or unlinked [83, 86]. Together, *T. reesei* has high RIP activities before premeiotic DNA synthesis, thus RIP strategy can apply to inactivate the gene expression for functional studies or strain improvement engineering.

## CRISPR/Cas9 system

The CRISPR/Cas (Clustered regularly interspaced short palindromic repeats-CRISPR associated) is an acquired immune system in bacteria and archaea to protect against invasion from bacteriophages and other foreign genetic elements [94, 95]. There are two classes of CRISPR/Cas systems. Class 1 CRISPR/Cas systems contain multiple Cas proteins that form an effector complex with crRNA (CRISPR RNA) to recognize and process the target. By contrast, Class 2 systems have a single, multidomain crRNA-binding protein to perform the analogous function with the entire Cas proteins of class 1 [96–98]. The class 2 CRISPR/Cas systems have been developed as a promising DNA or RNA editing tool in both prokaryotic and eukaryotic organisms because its effector complex contains only two components, Cas nuclease and artificially designed single guide RNA (sgRNA) that includes a specific crRNA fragment that pairs with the target and auxiliary trans-activating RNA (tracrRNA) that interacts with Cas nuclease and crRNA [99, 100].

**Fig. 5:**
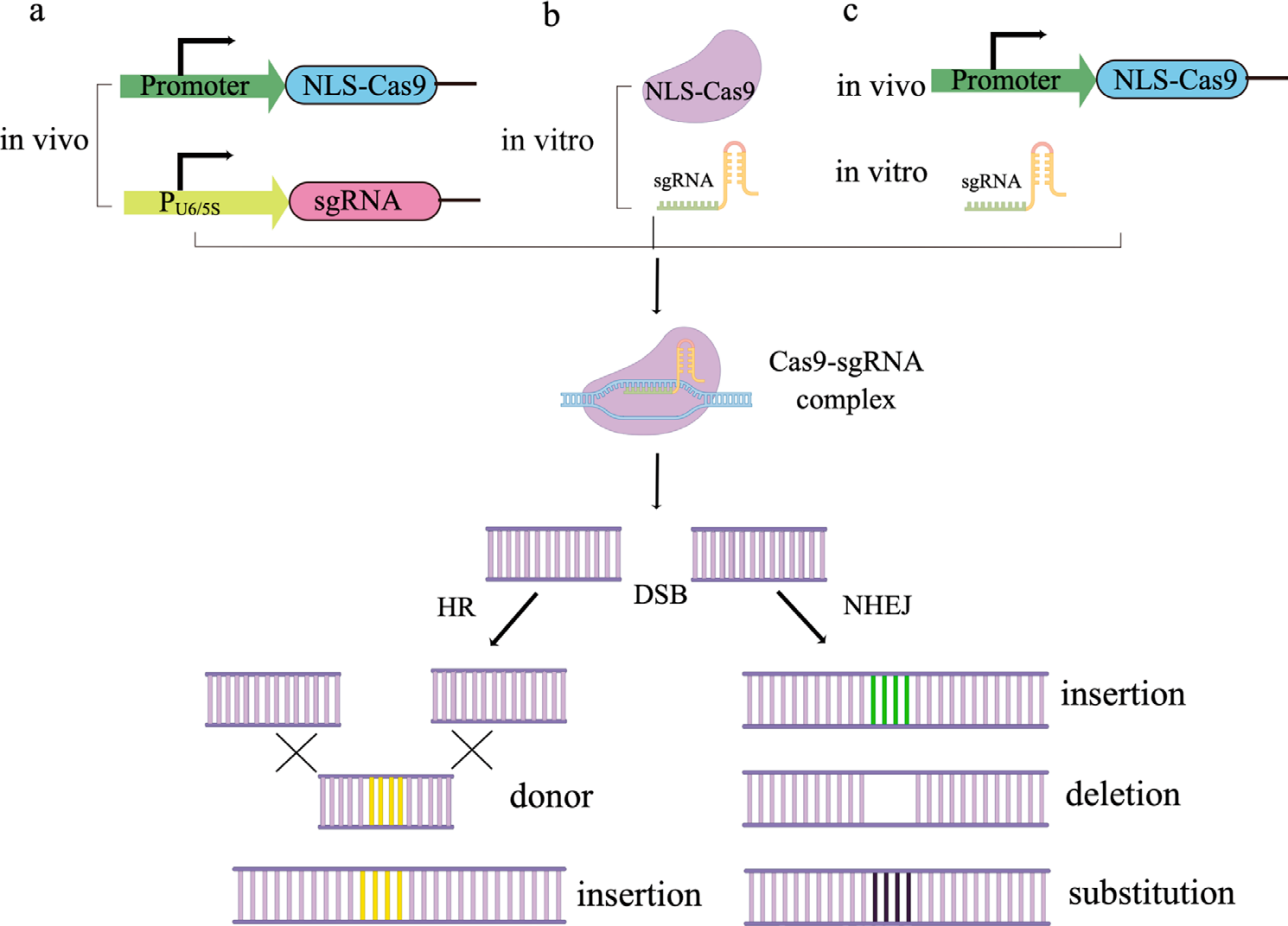
**Schematic diagram of the CRISPR/Cas9 system developed in*T. reesei***. (a) Two plasmids expressing NLS-Cas9 fusion protein and sgRNA were constructed, respectively, and were delivered together to cells. (b) The NLS-Cas9 protein and sgRNA were synthesized and assembled in vitro, and then they were transformed together into cells. (c) A plasmid expressing NLS-Cas9 fusion protein was constructed and delivered to cells. The sgRNA was prepared by in vitro transcription and transformed into host cells harboring the NLS-Cas9 expression cassette. The Cas9-sgRNA complex unwinds the double-stranded DNA (dsDNA) and the sgRNA binds to one of the DNA strands. Upon binding, the Cas9 nuclease cleaves both DNA strands upstream of the PAM sequence to form DSB, which is repaired either by the HR pathway or the NHEJ pathway

Up to now, the identified Cas nucleases of the Class 2 system are classed into three types (II, V, VI) which contain Cas9, Cas12, and Cas13 as an effector module to participate in DNA, DNA/RNA, and RNA editing, respectively [96]. Among them, the Cas9 nuclease from *Streptococcus pyogenes* (*Sp*Cas9) has been widely applied for genome editing in filamentous fungi [101, 102]. The efficient gene editing mediated by *Sp*Cas9 at DNA sequences requires an NGG protospacer adjacent motif (PAM) downstream target position [103]. In 2015, Liu et al. expressed a codon-optimized *Sp*Cas9 with an SV40 nuclear localization signal (NLS) under the control of the constitutive promoter P_*pdc*_ (pyruvate decarboxylase-encoding gene promoter) and inducible promoter P_*cbh1*_ (cellobiohydrolase I-encoding gene promoter), respectively, in *T reesei* [104]. Owing to the lack of identified RNA polymerase III-based promoters, they decided to synthesize the sgRNA by in vitro transcription and then deliver it to cells (Fig. 5). Once the *Sp*Cas9 and sgRNA form an effector complex, the *T. reesei* genome will generate DSBs at the appointed position determined by crRNA. Immediately, the DSBs can be repaired through the NHEJ pathway, which generates deletions, substitutions, or insertions near to cleavage site. As a result, the editing efficiency of *Sp*Cas9, whether its expression was controlled by P_*pdc*_ or P_*cbh1*_ promoter, reached 100% at the appointed position of the *ura5* proved by followed DNA sequencing [104]. Meanwhile, Liu et al. tested the HR efficiency by introducing an exogenous DNA fragment flanking the homology arms with different lengths in the presence of *Sp*Cas9-mediated DSBs. It is sufficient (> 93%) to achieve efficient HR even though only 200 bp homology arms were used [104].

In 2020, Wu et al. identified two *T. reesei* U6 small nuclear RNA (snRNA) gene promoters that include approximately 500 bp regions upstream of two U6 genes found by BLASTN using the *Myceliophthora thermophila* U6 snRNA gene as the template. The success of *ura5* gene editing wherein the sgRNA expression under the control of the U6 snRNA promoters offsets the lack of RNA polymerase III-based promoters for the *T. reesei* CRISPR/Cas9 system [105]. Afterward, Wang et al. identified a 5S ribosomal RNA (rRNA) gene in *T. reesei* by in silico analysis using the already defined 5S rRNA gene sequence of *Trichoderma virens* as a query and selected its promoter to express sgRNA. In this CRISPR/Cas9 system, the *lae1* coding region was edited at efficiency of 36.67% by using markerless donor DNA flanking 500 bp homology arms (Fig. 5) [106].

Although CRISPR/Cas9 system has vast application potential in a variety of species, from bacteria to humans, the construction of a host strain carrying Cas9 nuclease and/or sgRNA expression cassette is time-consuming and laborious. Moreover, the constitutive expression of Cas9 in host strain will may result in impaired growth of cells, DNA rearrangements, or off-target editing [107–109]. Hence, the transient transformation of in vitro prepared ribonucleoprotein (RNP) complexes consisting of Cas9 protein and sgRNA with or without donor DNA is an alternative safe method to avoid side effects [110, 111]. This strategy is not limited by the Cas9 expression level and sgRNA transcription. Furthermore, some integrational mutagenesis can be avoided during gene delivery [112]. In 2019, Hao et al. achieved 30% editing efficiency at the *T. reesei cbh1* locus by virtue of in vitro assembled Cas9/sgRNA complex, providing a fast and effective gene disruption strategy with the CRISPR/Cas9 system (Fig. 5) [107]. However, during the test of HR-mediated gene replacement, they observed that gene editing exhibits a low frequency by co-transformation of Cas9/sgRNA with a donor DNA in *T. reesei* [107]. Because of similar cases in other fungi, a possible reason is the much lower absorption efficiency of RNPs by fungal protoplasts [113]. To improve the RNPs delivery efficiency, Zou et al. modified the transformation strategy by adding the surfactant Triton X-100 to increase cell membrane permeability and prolong the incubation time during the protoplasts transformation process [114]. A 56.52% HR event of donor DNA with 20 bp homology arms at the vicinity of the cleavage site was found [114]. In addition, gene disruption was also achieved by this optimized RNP-mediated transformation without foreign donor DNA, although the efficiency was relatively low (7.37%) [114]. Compared with the intracellular expression of Cas9 and/or sgRNA, the transient transformation of RNPs could avoid the unwanted effects to host cells brought by the transgenic strategy. Nonetheless, further improvement of gene disruption efficiency using RNPs is still necessary to economize the screening time of correct transformants. Furthermore, many methods have been employed to break through the limitations caused by the off-target effects of *Sp*Cas9 and its rigorous requirement for the PAM sequence [115, 116]. These improvements could ensure gene editing at any position of the genome regardless of DNA contexts and subsequent off-target effects in both prokaryotic and eukaryotic organisms.

## Gene complementation and overexpression

In general, gene complementation was required to further verify the relevance between gene mutant and acquired phenotype. Gene complementation also needs a cassette that contains promoter, ORF, terminator, and selection marker. In *T. reesei*, a native promoter of mutated gene is more popular during gene complementation because this promoter can drive its expression in complementation strain to reach a similar level with wild type strain, which will account well for the gene functions [117]. In view of the most genes subjected to functional investigation are novel, sometimes it may be difficult to define their specific and functional promoter due to complicated gene structure in eukaryotic cells. Therefore, in some cases, common promoters from other genes of *T. reesei* with similar expression capacity to native promoter will be selected to finish the gene complementation [118, 119]. In a few cases, gene mutants severely affect the receptivity of exogenous DNA, resulting in failure of the next gene complementation [120]. Therefore, three independent transformants at least are necessary to exclude the possible effects of mutant cassette insertion into the genome [48].

Gene overexpression is also an important strategy to investigate gene functions by increasing the expression level of interested genes. Combinatory analysis of gene mutant and overexpression can provide more convincing evidences about biological functions of target genes [121]. For gene overexpression strategy in *T. reesei*, it seems to be similar to gene complementation. A key point is the researchers need to consider promoting the expression of target genes to a higher degree than its expression in control strain by virtue of promoter with high expression capacity. Some common promoter elements for gene complementation or overexpression in *T. reesei* are listed in Table 2. Besides, some engineered promoters are designed and developed based on properties of native promoter to enhance target gene expression in *T. reesei* [122, 123]. The common approach to modify the native promoter is adding activation element or deleting the repression element.

The selection of terminator in complementation/overexpression cassette is more flexible although it serves as a critical role in mRNA stability and translation. In *T. reesei*, terminators originating from cellulase genes are commonly used [14, 124]. Besides, some other terminators, such as *trpC* and *cox4*, are also used for gene expression [14, 15]. But until now, there is not much data available about the influence of the terminators on gene expression in *T. reesei*.

**Table 2:** Common promoters used in *T. reesei* [78, 125]

Promoter	Gene function	Remarks
**Constitutive**		
*cDNA1*	Unknown	Strong, commonly used
*pdc1*	Pyruvate decarboxylase	Highly expressed on glucose-containing media
*eno1*	Enolase	Highly expressed on glucose-containing media
*tef1*	Transcription elongation factor 1α	Medium strong, activity is lower than *cDNA1* promoter
*pki1*	Pyruvate kinase	Medium strength
*pgk1*	3-phosphoglycerate kinase	Medium strength
*gpd1*	Glyceraldehyde-3-phosphate dehydrogenase	Stable activity on D-glucose
**Tunable**		
*cbh1*	Cellobiohydrolase (Cel7A)	Inducible with cellulose, sophorose, and lactose. Repressible with D-glucose
*cbh2*	Cellobiohydrolase (Cel6A)	
*egl1*	Endoglucanase (Cel7B)	
*egl2*	Endoglucanase (Cel5A)	
*xyn1*	Xylanase I	Inducible with xylan and D-xylose (concentration dependent). Repressible with the D-glucose and D-xylose (concentration dependent)
*xyn2*	Xylanase II	Inducible with xylan, D-xylose (concentration dependent), xylobiose, cellobiose, and sophorose. Repressible with D-xylose (concentration dependent) and partly with D-glucose
*xyn3*	Xylanase III	Inducible with cellulose, L-sorbose and sophorose. Repressible with D-glucose and D-xylose (concentration dependent)
*tcu1*	Copper transporter	Repressible with copper
*tauD3*	TauD like dioxygenase	Repressible with L-methionine

## Conclusions

The filamentous fungus *T. reesei* not only holds ascendancy for carbohydrate-active enzymes (CAZymes) or recombinant protein production but also can be applied for direct conversion of waste plant biomass to valuable chemicals by metabolic engineering [126–128]. Clarifying the function of genes by genetic modification is an indispensable procedure for more achievements. We therefore outline the advances of the current genetic strategies in *T. reesei* for investigating gene functions, which will contribute to functional analysis of novel genes and construction of *T. reesei* cell factory. Some of the strategies mentioned in this review can also be beneficial to function investigation of genes in other filamentous fungi.

## Abbreviations

ORF: open reading frame
RIP: repeat-induced point mutation
UV: ultraviolet
ROS: reactive oxygen species
HR: homologous recombination
NHEJ: non-homologous end joining
DSBs: double-strand breaks
siRNA: small interfering RNA
RNAi: RNA interference
dsRNA: double-stranded RNA
shRNA: short hairpin-RNA
*cbh2*: cellobiohydrolase II gene
*cbh1*: cellobiohydrolase I gene
RT-qPCR: quantitative real-time polymerase chain reaction
SDS-PAGE: sodium dodecyl sulfate–polyacrylamide gel electrophoresis
SAGA: Spt-Ada-Gcn5 acetyltransferase
CRISPR/Cas: clustered regularly interspaced short palindromic repeats-CRISPR associated
crRNA: CRISPR RNA
sgRNA: single guide RNA
tracrRNA: trans-activating RNA
*Sp*Cas9: Cas9 nuclease from *Streptococcus pyogenes*
PAM: protospacer adjacent motif
NLS: nuclear logcalization sequence
P_*pdc*_: pyruvate decarboxylase-encoding gene promoter
P_*cbh1*_: cellobiohydrolase I-encoding gene promoter
snRNA: small nuclear RNA
rRNA: ribosomal RNA
RNP: ribonucleoprotein
dsDNA: double-stranded DNA
CAZymes: carbohydrate-active enzymes.
